# Are sympatrically speciating Midas cichlid fish special? Patterns of morphological and genetic variation in the closely related species *Archocentrus centrarchus*

**DOI:** 10.1002/ece3.2184

**Published:** 2016-05-20

**Authors:** Carmelo Fruciano, Paolo Franchini, Francesca Raffini, Shaohua Fan, Axel Meyer

**Affiliations:** ^1^ Department of BiologyChair of Zoology and Evolutionary BiologyUniversity of KonstanzUniversitätsstrasse 1078457KonstanzGermany; ^2^ School of Earth, Environmental & Biological SciencesQueensland University of TechnologyBrisbaneQld4000Australia; ^3^ International Max Planck Research School (IMPRS) for Organismal BiologyMax‐Planck‐Institut für OrnithologieAm Obstberg 178315RadolfzellGermany

**Keywords:** Adaptive radiation, geometric morphometrics, phylogeography, sympatric speciation

## Abstract

Established empirical cases of sympatric speciation are scarce, although there is an increasing consensus that sympatric speciation might be more common than previously thought. Midas cichlid fish are one of the few substantiated cases of sympatric speciation, and they formed repeated radiations in crater lakes. In contrast, in the same environment, such radiation patterns have not been observed in other species of cichlids and other families of fish. We analyze morphological and genetic variation in a cichlid species (*Archocentrus centrarchus*) that co‐inhabits several crater lakes with the Midas species complex. In particular, we analyze variation in body and pharyngeal jaw shape (two ecologically important traits in sympatrically divergent Midas cichlids) and relate that to genetic variation in mitochondrial control region and microsatellites. Using these four datasets, we analyze variation between and within two Nicaraguan lakes: a crater lake where multiple Midas cichlids have been described and a lake where the source population lives. We do not observe any within‐lake clustering consistent across morphological traits and genetic markers, suggesting the absence of sympatric divergence in *A. centrarchus*. Genetic differentiation between lakes was low and morphological divergence absent. Such morphological similarity between lakes is found not only in average morphology, but also when analyzing covariation between traits and degree of morphospace occupation. A combined analysis of the mitochondrial control region in *A. centrarchus* and Midas cichlids suggests that a difference between lineages in the timing of crater lake colonization cannot be invoked as an explanation for the difference in their levels of diversification. In light of our results, *A. centrarchus* represents the ideal candidate to study the genomic differences between these two lineages that might explain why some lineages are more likely to speciate and diverge in sympatry than others.

## Introduction

Sympatric speciation (i.e., divergence in the face of gene flow) has been controversial for a long time (Mayr 1963; Via 2001; Mallet et al. 2009). While even the existence of this mode of speciation has been subject of intense debate in the past (Mayr 1963; Smith 1966; Via 2001), today the focus of research has shifted towards asking which ecological conditions facilitate it (Via 2001; Coyne 2007; Bird et al. 2012). Theoretical and empirical studies are exploring the conditions at the genomic level which could promote sympatric speciation (Via 2001, 2012; Gavrilets et al. 2007; Michel et al. 2010; Via et al. 2012; Flaxman et al. 2014; Franchini et al. 2014); recent empirical studies also show that such a phenomenon might be more common than initially thought (Papadopulos et al. 2011).

Midas cichlids (*Amphilophus* spp.) in Nicaraguan crater lakes represent one of the few recognized cases of sympatric speciation (Bird et al. 2012). These fish inhabit the two largest Nicaraguan lakes (Lake Nicaragua and Lake Managua); these are old (early Pleistocenic; Kutterolf et al. 2007), shallow, and turbid. From these two lakes, founding populations colonized repeatedly and independently a number of geographically close crater lakes. Compared with the tectonic Lakes Nicaragua and Managua, these crater lakes are younger (Kutterolf et al. 2007), smaller, deeper, and filled with clear water. In the crater lakes, Midas cichlids have repeatedly and rapidly diverged in sympatry (Meyer 1990; Elmer et al. 2010b) into open‐water (limnetic) and bottom‐dwelling (benthic) species (Barluenga et al. 2006; Barluenga and Meyer 2010; Elmer et al. 2010b), as a consequence of the ecological opportunities that these crater lakes provided (e.g., the existence of open‐water and benthic habitats). So far, 11 new forms from the crater lakes have been formally described as new species, distinct from the generalist *A. citrinellus* of the source tectonic lakes. These fish have not only diverged in sympatry within Nicaraguan crater lakes, but they have also diverged in allopatry between their source (large tectonic lakes, Lakes Nicaragua and Managua) and derived crater lakes. The Midas cichlid endemic of crater lakes are morphologically (Klingenberg et al. 2003; Elmer et al. 2010b; Franchini et al. 2014) and genetically (Barluenga and Meyer 2004, 2010) distinct from each other and from the species of the tectonic lakes.

Among the crater lakes which harbor distinct species of the Midas cichlid group, lakes Apoyo and Asososca Managua host a relatively small number of other fish species (Waid et al. 1999); in particular, only one other cichlid species (*Parachromis managuensis*; a piscivorous fish which feeds on Midas cichlids). In contrast, Lake Xiloá hosts a rich community of cichlid fish. Apart from fish of the Midas cichlid complex (one limnetic and three benthic species; Recknagel et al. 2013), eight other cichlid species inhabit this lake (Waid et al. 1999). It is remarkable that, contrasting with the rapid diversification of Midas cichlids, there are no reports of either sympatric or allopatric differentiation in species belonging to other cichlid lineages in Nicaraguan crater lakes. The other cichlid fish inhabiting Lake Xiloá therefore belong to the same nominal species present both in the main lakes Managua and Nicaragua and in other central American environments. To date, however, no study on the intraspecific variation in these other cichlid fish from Nicaraguan lakes has been carried out. Such studies would reveal cryptic variation within or between lakes, if this ever existed. It is, then, possible that the apparent singularity of Midas cichlids' diversification is due to lack of intraspecific studies on the other cichlids living in Nicaraguan lakes.

Morphological and genetic divergence among geographically distinct locations is also often, but not always, reported (Walker 1997; Maderbacher et al. 2008; Fruciano et al. 2011a, 2011b, 2011c). This is connected to allopatric divergence and to the general question of what is responsible for the variation among clades in diversity and disparity (Collar et al. 2005; Ricklefs 2006; Rabosky 2009; Wagner et al. 2012; Morlon 2014; Hughes et al. 2015). Current theoretical models, indeed, propose a role for both extrinsic and intrinsic factors and their interaction in facilitating diversification and radiation (Bouchenak‐Khelladi et al. 2015; Donoghue and Sanderson 2015). Extrinsic causes include the variation of ecological parameters in space; intrinsic factors comprise both genetic architecture and phenotypic traits (Donoghue and Sanderson 2015; Seehausen 2015).

Therefore, comparing Midas cichlids with species belonging to other lineages would help to clarify if there is something special about Midas cichlids and their biodiversity. Specifically, if cichlids belonging to other lineages really did not diversify, why was the Midas cichlid lineage able to speciate both in sympatry and allopatry but the other lineages did not?

To address these questions, we studied another cichlid species living both in the large lakes and in Lake Xiloá: *Archocentrus centrarchus*. According to the latest molecular phylogenies of Neotropical cichlids, *A. centrarchus* is the cichlid of Nicaraguan crater lakes that is most closely related to the Midas cichlid complex (López‐Fernández et al. 2010). While the exact trophic habits of non‐Midas cichlid fish in Nicaraguan crater lakes are not known, studies in other locations show that among the fish inhabiting these lakes *A. centrarchus* has the most similar feeding niche to the one of Midas cichlids. Indeed, both *A. centrarchus* and *A. citrinellus* are considered “deep‐bodied vegetation‐dwelling invertebrate feeders” (Winemiller et al. 1995), as opposed to other species which are more herbivorous, piscivorous, or substrate diggers.

Here, we use a combination of different morphological and molecular datasets to explore the possibility that *A. centarchus* exhibits cryptic divergence within and among Nicaraguan lakes. If this variation were consistent across genetic and morphological datasets, this would suggest a previously unrecognized diversification of its lineage. In particular, we chose as morphological traits body and pharyngeal jaw shape. These are two very important traits, which probably played an important role during ecological speciation with gene flow in Midas cichlids in response to specialization to benthic and limnetic habitats (Barluenga et al. 2006; Elmer et al. 2010b, 2014; Franchini et al. 2014). Similarly, we analyzed variation of the mitochondrial control region and at 12 microsatellite *loci*. These are the same genetic *loci* that diverged between populations of Midas cichlids and which, together with other evidence, support sympatric speciation in Midas cichlids (Barluenga et al. 2006; Barluenga and Meyer 2010).

We hypothesized that if *A. centrarchus* has diverged in multiple forms within or across lakes, we should be able to retrieve a consistent signature of divergence across morphological and genetic data. We also hypothesized that if *A. centrarchus* has adapted to the crater lake environment, it should exhibit higher levels of morphospace occupation in crater lakes than in source lakes. Finally, we also tested the hypothesis that *A. centrarchus* and Midas cichlids both colonized crater lake Xiloá simultaneously, as a different timing of colonization might explain difference in diversification between Midas and non‐Midas cichlids. In fact, if *A. centrarchus* did not exhibit intraspecific divergence, one possible explanation would be that Midas cichlids colonized crater lake Xiloá earlier than *A. centrarchus*. If this was the case, Midas cichlids could have occupied multiple niches by diverging into benthic and limnetic species; thus, filling these niches before *A. centrarchus* could occupy them and diverge.

## Materials and Methods

A total of 71 *A. centrarchus* specimens were used in the present study (Table 1; Appendix S1). Fish were collected in Nicaraguan Lakes Managua and Xiloá in 2012 and photographed after collection. A fin clip was taken for molecular analyses, and the specimens were stored in ethanol. The lower pharyngeal jaws were later dissected from ethanol‐preserved specimens and photographed in a standardized fashion using a copy stand.

**Table 1:** Sample sizes of the different morphological and molecular datasets used in this study

Lake	Body shape	Pharyngeal jaw shape	mtDNA control region	Microsatellites
Managua	17	22	22	25
Xiloá	44	30	40	32
Total	61	52	62	57

### Morphometric analyses

**Figure 1:**
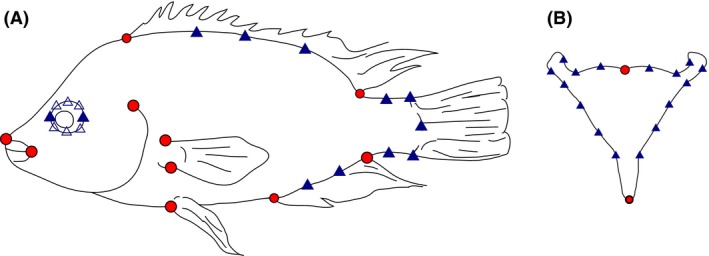
Configurations of points used in the morphometric analyses of body (A) and pharyngeal jaw (B) shape. Red circles = landmarks; filled blue triangles = semilandmarks; empty blue triangles = “helper” semilandmarks.

The configurations of points used in morphometric analyses of body and pharyngeal jaw shape (Fig. 1) comprised landmarks, semilandmarks, and “helper points.” “Helper points” are semilandmarks used to help the alignment of the other points, but that are later removed from the analysis as they do not provide additional information (Zelditch et al. 2004)). Points were digitized on body and pharyngeal jaw photographs using tpsDig 2.57 (Rohlf 2013). For a subset of specimens (about 1/4 of the total) presenting damage in one of the two pharyngeal jaw horns, we obtained estimates of the missing points by reflecting the corresponding points across the symmetry axis (Martínez‐Abadías et al. 2009; Couette and White 2010). The obtained configurations of points (*x*,* y* coordinates) were subjected to a generalized Procrustes analysis with sliding of semilandmarks (Bookstein 1997) in tpsRelW 1.54 (Rohlf 2007). Asymmetry was not of interest in the present study; therefore, all the subsequent analyses on pharyngeal jaws were performed on the symmetric component of shape variation (Klingenberg et al. 2002; Fruciano et al. 2011c). Allometry was controlled for, both in the case of body and pharyngeal jaw shape, by regressing shape variables on body centroid size and using regression residuals in subsequent analyses.

Differences between lakes in body and lower pharyngeal jaw shape were tested in MorphoJ 1.06b (Klingenberg 2011) using the permutational procedure based on Procrustes distances. Correct classification rates for discriminant analyses were also obtained through the leave‐one‐out cross‐validation procedure implemented in the software. To visualize variation and level of overlap between lakes in body and pharyngeal jaw morphology, we used between‐group principal component analysis (Boulesteix 2005). This ordination technique is increasingly used in geometric morphometrics (Firmat et al. 2012; Seetah et al. 2012; Franchini et al. 2014, 2016; Fruciano et al. 2014; Schmieder et al. 2015), as the ordinations do not exaggerate the extent of separation between groups, one of the typical drawbacks of the commonly used scatterplots of canonical variate scores (Mitteroecker and Bookstein 2011).

Levels of morphological integration (Olson and Miller 1958; Goswami and Polly 2010) in body and pharyngeal jaw for each of the two lakes were quantified obtaining bootstrap estimates (1000 bootstrap replicates) of the scaled variance of the eigenvalues (Young 2006), computed as the variance of eigenvalues divided by the squared total variance.

For each of the two morphological traits, we also performed an analysis of morphospace occupation (i.e., an analysis of the intraspecific variability in each of the two populations of *A. centrarchus*) using allometry‐corrected data. This analysis, in the spirit of other similar analyses of intraspecific variation at different sampling sites (Fruciano et al. 2014), uses three different multivariate estimators of variability (multivariate variance, mean pairwise Euclidean distance, and mean Euclidean distance from lake centroid). We obtained estimates for each of these statistics and tested for differences between the two lakes using MDA (Navarro 2003). The estimates were obtained by rarefaction to the smallest sample size through bootstrap resampling; the test for difference in morphospace occupation between lakes was performed using the BTailTest procedure implemented in MDA. The rationale for performing such analyses of morphospace occupation is that, even in the absence of divergence in distinct open‐water and bottom‐dwelling forms, *A. centrarchus* could adapt to the new crater lake environment by individually specializing along the benthic–limnetic continuum. If this were the case, we would then expect *A. centrarchus* from Lake Xiloá to occupy a larger morphospace than *A. centrarchus* from Lake Managua.

The significance of the covariation between body and pharyngeal jaw shape for the full dataset was tested in MorphoJ using the permutational procedure which employs Escoufier RV coefficient (Escoufier 1973) as a test statistic for the null hypothesis of complete independence between blocks of variables. The strength of the covariation between body and pharyngeal jaw was compared between lakes by computing rarified estimates of the RV coefficient (Fruciano et al. 2013) and by performing the permutation test (1000 permutations) for the difference in RV between two groups (Fruciano et al. 2013).

To assess the possible presence of cryptic clusters of individuals based on morphometric data without assuming a priori defined groups, a modified version of the algorithm proposed by Ezard et al. (2010) was used for both body shape and pharyngeal jaws and both pooling observations and analyzing lakes separately. Briefly, the algorithm consists in a dimensionality reduction step obtained by performing a principal component analysis and retaining only the subset of the principal components with highest explanatory power followed by a model‐selection based approach to identify the most supported partitioning in clusters. As in the Ezard et al. (2010) formulation and code, we used the broken stick as stopping rule (Jackson 1993) to identify the subset of principal components to retain and we employed the method based on Gaussian mixture models implemented in the R package *mclust* (Fritsch 2012) to identify the best partitioning in clusters. Differently from Ezard and colleagues who used robust principal component analysis in the dimensionality reduction step, here we use a standard principal component analysis on the covariance matrix, deemed more appropriate in our case based on preliminary tests on fish body and pharyngeal jaw shape interspecific variation on other cichlid species (C. Fruciano, unpubl. data).

### Molecular analyses

Genomic DNA was extracted using standard salt extraction protocols. A standard fragment of the mitochondrial control region was amplified using primers LProF (Meyer et al. 1994) and CIC3 (Elmer et al. 2013) and sequenced obtaining, after trimming flanking regions, a 974 bp sequence. Sequences were aligned using ClustalW (Larkin et al. 2007) and the alignment was refined manually. The observed variation in mitochondrial control region sequences was tested against the expected variation under the null hypothesis of neutral evolution using as test statistics *F*u's *F*s (Fu 1997) and Ramos‐Onsins and Rozas *R* ^2^ (Ramos‐Onsins and Rozas 2002), assessing their significance using the coalescent simulations (1000 simulated samples) implemented in Arlequin 3.11 (Excoffier et al. 2005) and DNAsp 5.0 (Librado and Rozas 2009), respectively. We chose these two tests because they are more powerful than a number of alternatives, although which one of them is more powerful depends on factors such as sample size (Ramos‐Onsins and Rozas 2002). The genetic differentiation between lakes was tested using the permutational procedure (1000 permutations) based on *F*_ST_ estimates implemented in Arlequin. To explore the relationships among haplotypes and how they were distributed among the two lakes, we constructed a median‐joining network (Bandelt et al. 1999) using the software Network 4.2 (Fluxus Technology, Ltd., Clare, United Kingdom), employing the “star contraction” option to reduce its complexity and weighing transversions three times more than transitions, as suggested for mitochondrial data (Rüber et al. 1999). For the sake of consistency with the morphometric analyses, we also used the clustering detection algorithm (see above) on the mitochondrial control region dataset. Therefore, we obtained Tamura and Nei (1993) genetic distances among individuals, we then performed principal coordinate analyses on genetic distance matrices, and we finally subjected the principal coordinate scores to the clustering algorithm described above.

Individuals were also genotyped at 12 microsatellite *loci*: UnH011 and Unh013 (McKaye et al. 2002), TmoM7 (Zardoya et al. 1996), Abur28, Abur82, Abur151, and Abur162 (Sanetra et al. 2009), Burtkit (Salzburger et al. 2007), M1M (=Acit1), M2 (=Acit2), M7 (=Acit3), and M12 (=Acit4) (Noack et al. 2000; Elmer et al. 2014). Microsatellites were amplified with fluorescent reverse primers (HEX, FAM, and NED dyes) and fragment length was analyzed with the internal size marker Genescan‐500 ROX (Applied Biosystems, Foster City, CA) on an ABI 3100XL Automated Sequencer (Applied Biosystems), and with GeneScan 3.7 and Genotyper 3.7 (Applied Biosystems) software packages. Micro‐checker 2.2.3 (van Oosterhout et al. 2004) was used to detect null alleles and scoring errors. The Bayesian approach implemented in Bayescan 2.0 (Foll and Gaggiotti 2008) was used in order to test each for neutrality. Global statistics of differentiation between lakes (*F*_ST_) were computed and tested for significance using Genetix 4.05 (Belkhir et al. 2004). A principal coordinate analysis was also performed in GenAlex 6.5 (Peakall and Smouse 2012) on the matrix of codominant genetic distances. To obtain a further representation of the relationships among individuals, we also used the method based on graph theory implemented in the software EDENetworks 2.18 (Kivelä et al. 2015) to construct a network starting from pairwise genetic distances between individuals and using the software's automatic thresholding algorithm. Finally, to investigate the presence of genetic clusters either in the pooled sample or in the samples from each lake, we applied two methods: the Bayesian clustering method implemented in the software Structure 2.3.4 (Pritchard et al. 2000) choosing the appropriate number of clusters with the Evanno approach (Evanno et al. 2005), and the method implemented in the software GeneClass 2.0 (Piry et al. 2004), which computes the probability that each individual belongs to each reference lake. Structure was run with a burn‐in period of 100,000 steps followed by 1,000,000 Markov chain Monte Carlo (MCMC) iterations. Ten independent runs were performed using an admixture model and allele frequencies correlated, as these parameters are recommended for detecting genetic structure when closely related populations are involved (Falush et al. 2003). GeneClass was run on 10,000 simulated individuals with an assignment threshold set at 0.05.

### Combined analyses of morphometric and genetic data

If adaptive divergence were occurring in *A. centrarchus*, this would produce concordant signals in genetic and morphometric data (i.e., in morphometric data as a consequence of specialization, in genetic data because of a reduction in gene flow; this is the situation encountered in Midas cichlid fish; e.g., Elmer et al. 2014). To test this hypothesis quantitatively, we measured the concordance among clustering approaches applied on different datasets. This was obtained by computing the adjusted Rand index (Hubert and Arabie 1985) on the observations overlapping between datasets in each pairwise comparison and testing its significance using a recently suggested permutational procedure (Qannari et al. 2014). The Rand index (Rand 1971) is an index that is expected to take the value of zero when two partitions of the same observations do not agree at all and one when the two partitions agree completely. The adjusted Rand index is a modification of the Rand index, which corrects the latter for chance and ensures a value of zero in the case of random partitions. Therefore, in the case of adaptive divergence revealed by both genetic and morphological data, we would expect the value of this index to be close to one and statistically significant.

### Timing of colonization

We tested the null hypothesis that the colonization of crater Lake Xiloá occurred simultaneously for both Midas cichlids and *A. centrarchus* using a comparative phylogeographic approach. In particular, we used msBayes 20081106 (Hickerson et al. 2007), in the same spirit as previously done (Elmer et al. 2013) for Midas cichlids and *Hypsophrys nematopus*. The msBayes software pipeline uses approximate Bayesian computation to test the null hypothesis of simultaneous divergence across lineages spanning a common geographic barrier (Hickerson et al. 2006). In fact, disparate levels of divergence across the same barrier might be a mere consequence of biological phenomena (such as variation in mutation or demographic parameters) rather than a different divergence time. msBayes overcomes this issue by incorporating population genetic parameters in a hierarchical model, which estimates jointly lineage‐specific parameters and parameters shared among lineages (called “hyperparameters”). This allows integrating uncertainty in parameter estimation, thus obtaining a more reliable estimate of the hyperparameter “psi” (number of distinct divergence events across lineages). We used this pipeline on a mitochondrial control region dataset to test for simultaneous divergence between source (Managua) and derived (Xiloá) lake in Midas cichlids and *A. centrarchus*. To this aim, we combined the sequences of this study with 370 published mitochondrial control region sequences of Midas cichlids from Lakes Managua and Xiloá (Barluenga and Meyer 2004, 2010; Bunje et al. 2007; Elmer et al. 2010a; Geiger et al. 2010) (Appendix S2). For this analysis, sequences from both *A. centrarchus* and Midas cichlids and from both lakes were pooled, aligned in ClustalW (Larkin et al. 2007) and trimmed to a common length of 711 bp. Prior to being subjected to the msBayes pipeline, sequences were then realigned by species. To test different migration scenarios, we performed the analysis using the upper bound for migration rate at zero and at 0.5.

## Results

### Morphometric analyses

**Figure 2:**
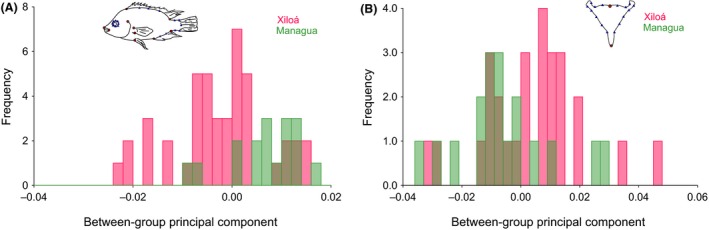
Scores along the between‐group principal component of body (A) and pharyngeal jaw (B) shape.

The average body shape of *A. centrarchus* is not significantly different between Lakes Managua and Xiloá (Procrustes distance 0.009, *P* = 0.49, 27.87% cross‐validated correct classification). After correcting for allometric variation, pharyngeal jaw shape is not significantly different between lakes (Procrustes distance 0.01, *P* = 0.1, 33.33% cross‐validated correct classification). Scores of individuals along the first between‐group principal component in both datasets confirm an extremely high degree of overlap in morphology between the two lakes (Fig. 2).

The strength of morphological integration is very similar in the two lakes for both body (scaled variance of eigenvalues, Managua = 0.004 Xiloá = 0.0035) and pharyngeal jaws (Managua = 0.0112 Xiloá = 0.0115). For both body and pharyngeal jaw shape, the distributions of the bootstrap estimates are largely overlapping between lakes, thus suggesting no difference between lakes in the levels of morphological integration.

In a similar fashion, levels of morphospace occupation in *A. centrarchus* were very similar between lakes for all three multivariate descriptors of disparity (Table 2) and never significantly different (BTailTest; *P* > 0.05 in all cases).

**Table 2:** Levels of morphospace occupation in each lake and for each trait. For each estimator, the mean and standard deviation obtained through rarefaction at the smallest sample size are provided

Sample	Multivariate variance	Mean pairwise Euclidean distance	Mean Euclidean distance from lake centroid
Body shape – Xiloá	0.001067 ± 0.000067	0.044773 ± 0.001382	0.031647 ± 0.001000
Body shape – Managua	0.001077 ± 0.000116	0.044204 ± 0.002452	0.031200 ± 0.001730
Pharyngeal jaw shape – Xiloá	0.000476 ± 0.000101	0.027567 ± 0.002871	0.019055 ± 0.001987
Pharyngeal jaw shape – Managua	0.000428 ± 0.000086	0.026487 ± 0.002661	0.018490 ± 0.001945

The covariation between body and pharyngeal jaw shape in the full sample is not significant (RV = 0.14, *P* = 0.2). The strength of covariation between body and pharyngeal jaw shape is not different between the two lakes (rarified at the same sample size of 15, RV = 0.395 ± 0.1 for the Managua sample and RV = 0.393 ± 0.1 for the Lake Xiloá sample; *P* = 0.94).

In the analysis of both body and pharyngeal jaw shape pooling all observations, the algorithm for cluster detection identified a single cluster. The same result was obtained analyzing the body shape for only the fish from Lake Managua and four clusters were identified analyzing the body shape of fish from Lake Xiloá. Three of them, however, contained only one observation each. For pharyngeal jaw shape, the analysis of the pooled sample identified two multivariate clusters, not corresponding to lakes. The same analysis performed on fish from each lake identified seven clusters for Lake Managua and two clusters for Lake Xiloá.

### Molecular analyses

**Figure 3:**
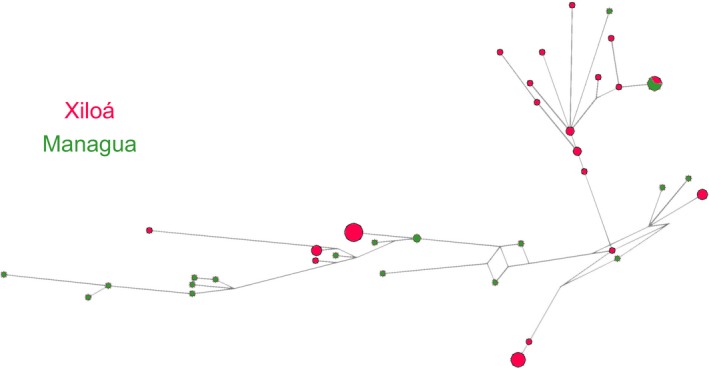
Mitochondrial control region median‐joining network. The size of the circles is proportional to the number of individuals represented.

The Ramos‐Onsins and Rozas *R* ^2^ was not significant in both lakes (Xiloá, *R* ^2^ = 0.1323, *P* = 0.78; Managua, *R* ^2^ = 0.1429, *P* = 0.74). On the other hand, *F*u's *F*s was significant only in the sample from Lake Managua (*F*s = −10.08, *P* < 0.001), but not in the one from Lake Xiloá (*F*s = −1.37, *P* = 0.33). The permutational procedure based on *F*_ST_ estimates suggested low but significant differentiation (*F*_ST_ = 0.063, *P* = 0.007) in mitochondrial control region sequences between the two lakes. The median‐joining network (Fig. 3) shows that haplotypes do not cluster in groups according to lake; rather, there are two main haplogroups containing haplotypes from both lakes. Further, there is no sign of “star‐like” genealogies, expected in the case of recent demographic expansion. The clustering algorithm identified seven clusters when applied to the full mitochondrial control region dataset, four clusters in each case when analyzing data by lake.

**Figure 4:**
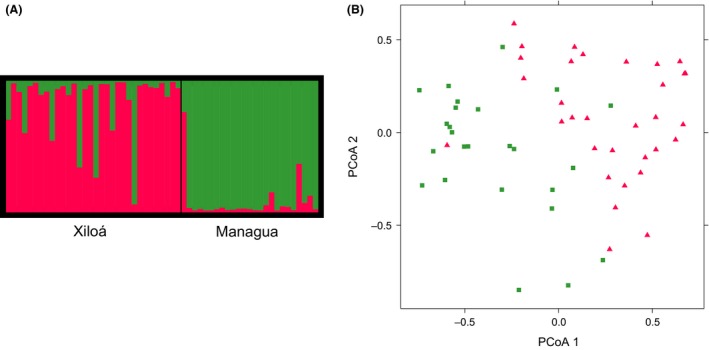
Analysis of microsatellite data. (A) Structure plot. (B) Scores along the first two principal coordinates (explaining, respectively, 11.63% and 8.82% of total variance) based on codominant genetic distances.

No sign of null alleles and directional selection were detected in the panel of 12 microsatellites employed in this study. The analysis of this *A. centrarchus* microsatellite dataset revealed a low but highly significant genetic differentiation between lakes (global *F*_ST_ = 0.07, *P* < 0.001). The network representation of the relationship among individuals based on microsatellite frequencies (Appendix S3) shows a low degree of overlap between individuals from the two lakes. This is confirmed by an inspection of the plot of the scores along the first two principal coordinates (Fig. 4), which shows two relatively clear clusters of individuals belonging to each lake with a few admixed individuals. The results of the two clustering approaches we used on the microsatellite dataset further confirm this pattern. In fact, the appropriate number of clusters chosen with the Evanno method is two (Appendix S4) and the Structure analysis (Fig. 4) reveals the existence of a few admixed individuals between lakes. In the GeneClass analysis, for all individuals the highest assignment probability is always obtained for the lake where they were sampled (Appendix S5). When performing the Structure analysis within each lake, no genetic clustering was observed (data not shown).

### Combined analyses of morphometric and molecular data

Our quantitative approach based on the adjusted Rand index revealed very poor overlap in the clustering of observations using different morphometric and genetic markers (Appendix S6), with values of the adjusted Rand index always low. Except in the case of the comparison of clustering of mtDNA and pharyngeal jaw shape, the adjusted Rand index is always nonsignificant. Even applying clustering methods to datasets of the same kind (i.e., both genetic or both morphometric) we find different partitions.

### Timing of colonization

**Figure 5:**
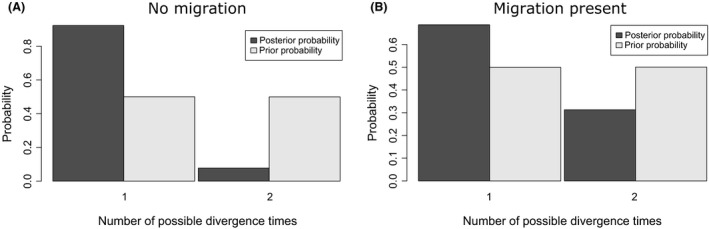
Results of the msBayes pipeline. The pipeline was run assuming equal prior probabilities for a single or two different divergence times between species. (A) No migration, (B) presence of migration.

The analyses performed with the msBayes pipeline produced globally concordant results, irrespective of the migration rates. In fact, in both cases, the posterior probability of a simultaneous divergence between lakes in the two species (i.e., a single divergence time for both species) was markedly higher than the posterior probability of the alternative scenario (i.e., two different divergence times for the two species; Fig. 5). This was particularly evident in the analysis with no migration. This is expected as migration can, obviously, obscure the signal of genetic isolation. However, when migration is a confounding factor in the analysis, this is reflected in the incorrect support of the temporal discordance in divergence between species.

## Discussion

We show that *A. centrarchus* does not exhibit any intraspecific divergence consistent across different genetic and morphometric datasets. In fact, not only did we not find evidence for multiple clusters (either genetic or morphological) within crater lake Xiloá (which would have suggested sympatric divergence in *A. centrarchus*), but also we found only marginal genetic differentiation between the source population from Lake Managua and that of the younger crater lake Xiloá population. Although there are significant differences between the two lakes in mitochondrial control region sequences, the value of *F*_ST_ (0.063) is much lower than the ones previously reported for Midas cichlids. Indeed, between *A. citrinellus* from Lake Managua and the three described Midas cichlids from Lake Xiloá the *F*_ST_ values range between 0.154–0.223 (Barluenga and Meyer 2010). On the other hand, using microsatellites we find in *A. centrarchus* significant levels of divergence between Lakes Managua and Xiloá, similar to those reported for Midas cichlids (*F*_st_ values range 0.059–0.078) (Barluenga and Meyer 2010). Interestingly, both the neutrality tests do not reject the null hypothesis of sequence variation conforming to a neutral model in Lake Xiloá. This clearly contrasts with the situation in Midas cichlids, where departures from neutrality (including in crater lake Xiloá) have been related to population expansion or selective sweeps (Barluenga and Meyer 2010). Morphometric analyses for both body and pharyngeal jaw shape reveal no significant differences between lakes, with very low correct classification rates for discriminant analyses and extensive overlap in between‐group principal component scores.

Overall, we interpret the observed mild genetic differentiation between lakes as a mere effect of neutral drift, not accompanied by a differentiation in morphology that would otherwise be suggestive of local adaptation. In fact, we fail to find any significant difference between lakes in the two morphological traits we studied. This finding holds true whether we analyze trait mean (tests for difference in means are not significant and there is an extensive overlap in between‐group principal component scores), trait disparity (very similar between lakes), trait integration (levels of integration are very similar), or levels of covariation between traits (not significantly different between lakes and, in general, not significant). Not only, then, we do not find morphological differentiation between lakes, but we do not observe patterns indicative of individual‐level adaptation fueled by the availability of new ecological niches (i.e., increase of morphospace occupation in the crater lake population), either.

The uniformity in morphology between the two populations of *A. centrarchus* studied here is surprising and unusual. In fact, morphological divergence between allopatric populations (not necessarily implying speciation) is commonly reported as a consequence of local adaptation and/or phenotypic plasticity, even in the presence of gene flow and among geographically close locations (Walker 1997; Klingenberg et al. 2003; Maderbacher et al. 2008; Elmer et al. 2010b; Fruciano et al. 2011b, 2011c).

We also tested for patterns of differentiation within lakes shared across different morphological markers. Using different approaches, we fail to identify any partition of the observed individuals consistent across different datasets. Our results, therefore, suggest a lack of sympatric differentiation in *A. centrarchus*.

## Conclusions

The current taxonomic status of *A. centrarchus* in Nicaraguan lakes (a single described species) is not due to lack of studies on intralineage variation (i.e., it is not due to ascertainment bias). The lack of both sympatric and allopatric divergence in *A. centrarchus* in the same lakes where the well‐known sympatric Midas cichlids have been described is an important finding. *A. centrarchus* and Midas cichlids represent closely related lineages with relatively similar ecological niches so, if one of the lineages underwent rapid differentiation but the other did not, understanding the causes of this difference in levels of differentiation will inform us on what are the factors that facilitate sympatric speciation.

By performing a comparative phylogeographic analysis, we also show that *A. centrarchus* and Midas cichlids have probably colonized crater Lake Xiloá at the same time. Therefore, although the resolution of a single marker in correctly resolving the time of colonization is somewhat limited, a different timing of colonization between Midas cichlids and *A. centrarchus* cannot be invoked as a parsimonious explanation for the difference in diversification rates between the two lineages. These results agree with a previous comparison of Midas cichlids with *Hypsophrys nematopus*, another cichlid which inhabits Lake Xiloá (Elmer et al. 2013). However, although Lake Managua is considered the source lake for Lake Xiloá, Elmer et al. (2013) did not study any sample from Lake Managua as *H. nematopus* is probably absent from this lake.

The similar levels of morphospace occupation between ancestral and derived populations of *A. centrarchus* can be explained hypothesizing that competition with Midas cichlids in the crater lake environment prevented *A. centarchus* from adapting to the new limnetic niche.

Collectively, our results open up new avenues of research investigating why Midas cichlids speciated copiously in the same lakes where other lineages, and in particular *A. centrarchus*, did not. Any possible explanation of the different patterns of diversification between Midas and non‐Midas cichlids in the Nicaraguan lakes is at this stage speculative. Nevertheless, our analyses of covariation between body and pharyngeal jaw show a lack of significant covariation (i.e., body shape and pharyngeal jaw are relatively independent in *A. centrarchus*). On the other hand, these two adaptive traits (the very traits that have diverged in sympatry in Midas cichlids) show significant covariation in Midas cichlids (C. Fruciano in prep.). Perhaps most importantly, in Midas cichlids body and pharyngeal jaw shape do not segregate independently and they have at least one overlapping QTL region (C. Fruciano, P. Franchini, V. Kovacova, K.R. Elmer, F. Henning, A. Meyer under rev.). Recent theoretical models emphasize the importance of pleiotropy and linkage in facilitating speciation (Flaxman et al. 2014) and could provide an useful framework that should be investigated in further research. Are the genetic bases of body and pharyngeal jaw shape independent in *A. centrarchus* and other non‐Midas cichlids from Nicaraguan crater lakes? We believe that it would be interesting to investigate this issue using comparative genetic and genomic studies, conducted in a phylogenetic context.

## Data Accessibility

Mitochondrial control region sequences have been deposited in GenBank under accessions KX227392‐KX227453. The remaining data is available from the Dryad Digital Repository: http://dx.doi.org/10.5061/dryad.vt968

## Conflict of Interest

None declared.

## Supporting information


**Appendix S1.** Detailed breakdown of the individual specimens used in this study.

**Appendix S2.** Genbank accession numbers of the Midas cichlid sequences used in the comparison of the timing of colonization between Midas cichlids and *A. centrarchus*.

**Appendix S3.** Network obtained with the approach implemented in EDENetworks using pairwise genetic distances in the microsatellite dataset.

**Appendix S4.** Line plot showing the mean, across the 10 independent runs of the Structure analysis, of (A) estimated log probability of data (lnP(D); bars represent standard deviation) and (B) of the delta *K* statistic (Evanno et al. 2005) for different *K* (number of genetic clusters).

**Appendix S5.** Results of the analysis performed in GeneClass.

**Appendix S6.** Overlap of the clustering obtained using different genetic and morphometric traits.
