# Supplementary material for: Are sympatrically speciating Midas cichlid fish special? Patterns of morphological and genetic variation in the closely related species Archocentrus centrarchus
Source: Ecol Evol. 2016 May 20;6(12):4102–14. doi: 10.1002/ece3.2184 (PMC4877357; doi:10.1002/ece3.2184)
Supplement: Supplementary file 2 — Appendix S2. Genbank accession numbers of the Midas cichlid sequences used in the comparison of the timing of colonization between Midas cichlids and A. centrarchus. [file ECE3-6-4102-s002.docx]

AY567244-AY567265

EF157327-EF157331

EF157374-EF157394

EF157477-EF157538

EF157573

EF219222-EF219228

EF219233-EF219234

EF219236

EF219238-EF219243

EF219246

EF219253-EF219265

EF219267

EF219271-EF219272

GU017062-GU017090

GU355867-GU355891

HM183718-HM183726

AY567307-AY567375

AY567411-AY567470

GU355793-GU355803

HM183727-HM183741

HM204814-HM204822
