# Supplementary figures and images for: Are sympatrically speciating Midas cichlid fish special? Patterns of morphological and genetic variation in the closely related species Archocentrus centrarchus
Source: Ecol Evol. 2016 May 20;6(12):4102–14. doi: 10.1002/ece3.2184 (PMC4877357; doi:10.1002/ece3.2184)

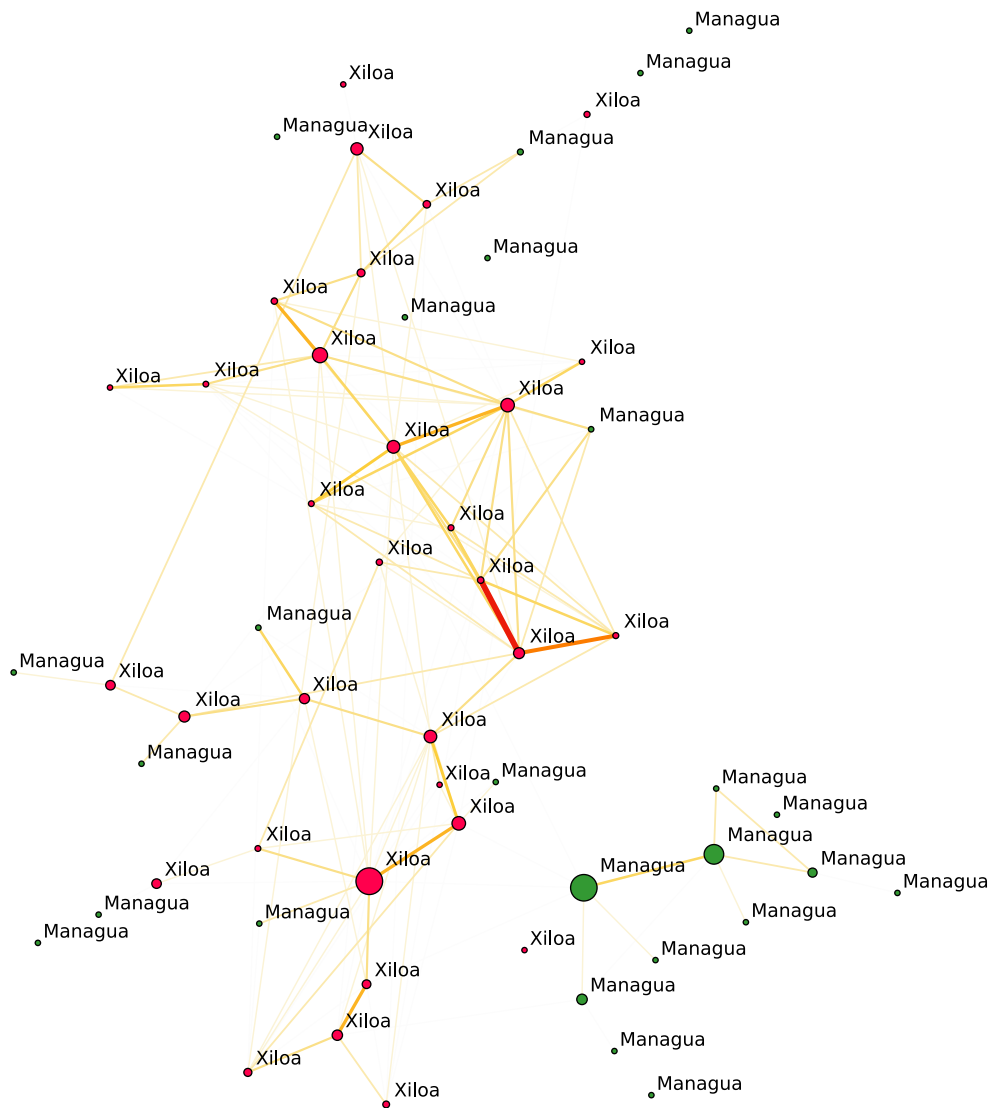

Supplement: Supplementary file 3 — Appendix S3. Network obtained with the approach implemented in EDENetworks using pairwise genetic distances in the microsatellite dataset. [file ECE3-6-4102-s003.pdf]

a

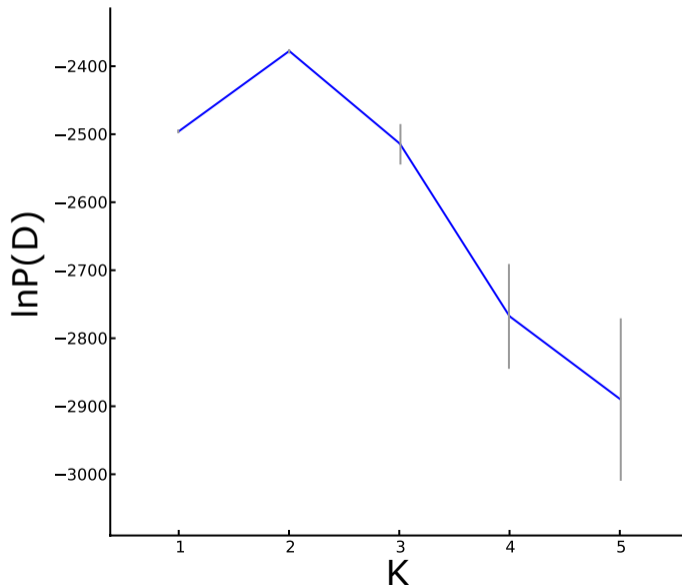

b

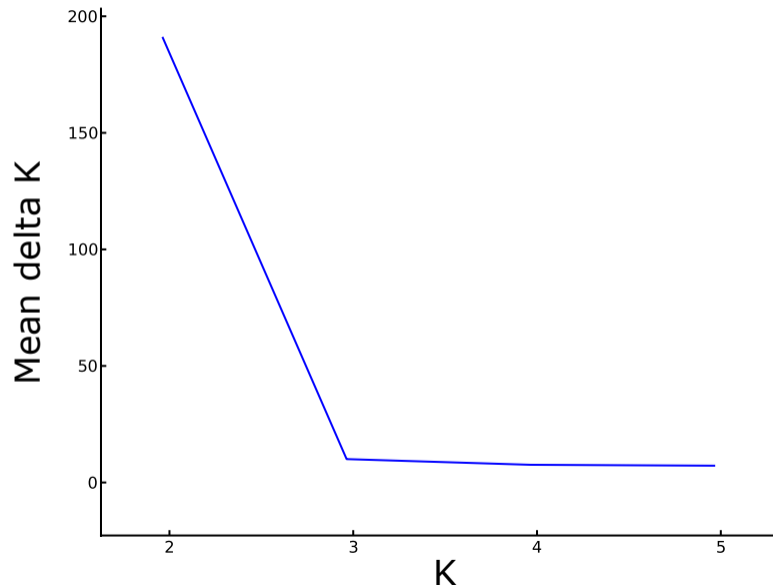

Supplement: Supplementary file 4 — Appendix S4. Line plot showing the mean, across the 10 independent runs of the Structure analysis, of (A) estimated log probability of data (lnP(D); bars represent standard deviation) and (B) of the delta K statistic (Evanno et al. 2005) for different K (number of genetic clusters). [file ECE3-6-4102-s004.pdf]
